# Perspectives From the Cardiovascular Health Council

**Published:** 2008-03-15

**Authors:** Miriam M Patanian

**Affiliations:** Cardiovascular Health Council, Heart Disease and Stroke Prevention Program, Washington State Department of Health

The Cardiovascular Health (CVH) Council is delighted to have this issue of *Preventing Chronic Disease* focus on the first and third leading causes of death in our nation — heart disease and stroke. This issue highlights some of the work being done nationally and by states and communities to prevent and manage cardiovascular disease. The authors in this issue encourage us to renew our commitment to addressing the nationwide epidemic of heart disease and stroke by intervening at all levels — prevention, risk reduction, emergency response, medical care, and rehabilitation.

## About the CVH Council

The CVH Council, housed within the National Association of Chronic Disease Directors, primarily comprises managers and staff of heart disease and stroke prevention programs in state health departments. Our vision is for all states and territories to be able to contribute effectively to a heart-healthy, stroke-free nation. The CVH Council serves as a national voice for the states and provides leadership and expertise on eliminating heart disease and stroke at the state and national levels. We link state, territorial, and tribal program directors and others so they can work collectively to promote cardiovascular health. We also develop partnerships with affiliates, private and public associations, and industry. We exchange ideas, strategies, materials, and policies to improve comprehensive public health programs aimed at cardiovascular diseases and their risk factors and advocate for legislation, policies, and programs to reduce these health problems. Finally, we comment and make recommendations on issues raised by federal agencies and members of the council or considered important by the council.

The CVH Council's officers, steering committee, and other committees and work groups carry out its work:

- The Advocacy and Policy Committee collaborates with partners to ensure that cardiovascular health issues are represented at all levels of government and sets the council's legislative agenda. The committee works to connect CVH Council members with states that have policies or legislation related to heart disease, stroke, and their risk factors. A work group established by this committee explores strategies to influence national initiatives to address heart disease and stroke risk factors.
- The Capacity Building Committee develops training programs and mentors state programs in cardiovascular health and program management. This committee has established a data work group to oversee several special projects and the CVH Council's internal evaluation.
- The Communications Committee informs council members on internal and external activities related to cardiovascular health.
- The Internal Council Organization Committee, whose priorities are membership development and council operations, maximizes resources to ensure that the council functions innovatively, efficiently, and effectively. The committee recruits state representatives from within and reviews operating guidelines, the strategic plan, and funding sources annually.
- The Partnerships Committee identifies and collaborates with external groups, organizations, and individuals to advocate for, develop, and implement cardiovascular health programs and policies. This committee, in partnership with the Advocacy and Policy Committee, established a work group to provide training for American Heart Association staff to ensure that volunteers understand and value the work of state programs.

## State Contributions to This Themed Issue

In this issue of *Preventing Chronic Disease*, the state programs show the broad spectrum of activities being carried out nationwide. Further opportunities for these programs are described in articles outlined in Brownstein's editorial (1). Several of the priority areas for states that have been outlined by the Centers for Disease Control and Prevention's (CDC's) Division for Heart Disease and Stroke Prevention in the National Center for Chronic Disease Prevention and Health Promotion are also addressed in this issue:

- **Control high blood pressure and high blood cholesterol.** State programs are working in these two priority areas in health care, community, and worksite settings, and two articles from CDC authors address these issues (2,3).
- **Increase knowledge of stroke signs and symptoms and the need to call 9-1-1.** Jurkowski et al (4) describe an evaluation of a media campaign in a four-county area of New York about the signs and symptoms of stroke and find a significant delay in seeking treatment for acute stroke. Having a history of seeking medical care emerged as a predictor for not seeking care for acute stroke. This article conveys the need for educational efforts to emphasize the seriousness of stroke symptoms and the need for emergency response. Wall et al (5) describe an evaluation of the stroke awareness animation from the Stroke Heroes Act FAST campaign in Massachusetts and find it to be effective in increasing and sustaining knowledge of stroke signs and symptoms.
- **Improve emergency response.** In the last several years, more states have begun to focus on emergency response to acute cardiac and stroke events. A common first step is to assess the current system, as Tsai (6) did with the Minnesota Stroke Partnership. Its survey of emergency medical services (EMS) directors and hospitals identified several strengths of the system, including a perception that EMS dispatchers considered stroke an emergency event, existence of policies to notify the destination hospital of an incoming patient suspected to be experiencing a stroke, and a perception that hospital staff attend to these patients immediately. Recommendations for improvements included standardizing prehospital assessment of these patients, developing and implementing standard emergency department protocols and standing orders, developing inpatient care protocols and pathways, and providing training in best practices to ensure the highest quality of care for patients with stroke.
- **Improve quality of care.** Meyer et al (7) describe how Maine's CVH and EMS programs have partnered to use prehospital data to plan and evaluate the state's heart disease and stroke program. This article offers several descriptive statistics about the data set, including the rate of transport runs for stroke and cardiac events and time-to-scene and overall transport times. Maine's CVH and EMS programs have formed the Maine HeartSafe Community Initiative, which recognizes local EMS services for their efforts and provides awareness and educational opportunities about heart disease and stroke. Next steps include linking EMS data to emergency department and hospital data sets for a more complete picture of the quality of care provided to patients with acute myocardial infarction or stroke.

All of these articles focus on work in health care and community settings. Brissette et al (8) describe a statewide, population-based worksite assessment in New York. Findings suggest that worksites with policy and environmental supports for primary prevention efforts, such as healthy eating, physical activity, and tobacco control, are likely to have secondary prevention efforts, such as screening for high blood pressure and high cholesterol. The take-away message is that more efforts are needed to promote policy and environmental supports for cardiovascular health, particularly among small and medium-small employers. The authors also suggest the importance of having a wellness committee or coordinator to promote and sustain wellness efforts in the workplace. Another article addresses tools available for the workplace (3).

## Challenges and Opportunities

As evidenced by the articles in this themed issue, state programs are working hard to prevent and manage heart disease and stroke. Interventions in many states — both funded and not yet funded by CDC — are having a positive impact on cardiovascular disease. A key area of focus for the CVH Council is to help secure adequate funding so all states and territories can fully address heart disease and stroke. We will continue to strive for this goal to fulfill our vision of all states and territories contributing effectively to a heart-healthy, stroke-free nation.

That heart disease and stroke are the first and third leading causes of death nationwide is not acceptable. If we as a nation collectively use the tools we have to prevent and manage these conditions, we could offer our population a decreased burden of cardiovascular risk and disease and, thus, an improved quality of life.
